# Iridocorneal angle imaging of a human donor eye by spectral-domain optical coherence tomography

**DOI:** 10.1038/s41598-023-37248-0

**Published:** 2023-08-24

**Authors:** Shangbang Luo, Guy Holland, Reza Khazaeinezhad, Samantha Bradford, Rohan Joshi, Tibor Juhasz

**Affiliations:** 1 Department of Biomedical Engineering, University of California, Irvine, Irvine, CA 92697 USA; 2 Department of Ophthalmology, University of California, Irvine, Irvine, CA 92697 USA; 3 ViaLase Inc., Aliso Viejo, CA 92656 USA

**Keywords:** Biomedical engineering, Biophotonics, Optical imaging

## Abstract

Iridocorneal angle (ICA) details particularly the trabecular meshwork (TM), Schlemm’s canal (SC), and collector channels (CCs) play crucial roles in the regulation of the aqueous outflow in the eyes and are closely associated with glaucoma. Current clinical gonioscopy imaging provides no depth information, and studies of 3D high-resolution optical coherence tomography (OCT) imaging of these structures are limited. We developed a custom-built spectral-domain (SD-) OCT imaging system to fully characterize the angle details. Imaging of a human cadaver eye reveals the visibility of details in the TM/SC/CC region via a ’crossline’ scanning and a series of image processing. This shows that ICA imaging can be used for preoperative glaucoma inspections in the clinical setting with the proposed prototype.

## Introduction

Glaucoma is the leading cause of irreversible blindness and is responsible for the second most common cause of visual loss in the world. Approximately 64.3 million people worldwide were affected in 2013, and this number is estimated to increase from 76.0 million in 2020 to 111.8 million in 2040^1^. Glaucoma is a neuropathy disease characterized by optic nerve damage or optic disc cupping and indicated by high intraocular pressure (IOP), which is caused by increased resistance to the normal outflow of aqueous humor (AH) from the eye^2^. The AH secreted by the ciliary body drains into the posterior chamber, then travels through the pupil into the anterior chamber (AC). At the ICA where the iris and cornea meet, the AH exits the eyes through two different outflow pathways: conventional and uveoscleral outflow pathways. Only 10% of the AH flows to the ciliary body surface and iris root towards the surrounding veins and tissues in the uveoscleral outflow pathway^3^. While the majority of the remaining AH fluid passes through the trabecular meshwork (TM) into Schlemm’s canal (SC). From there the fluid drains into a series of collector channels (CCs) and is finally absorbed by the episcleral veins^4^. The TM is a porous, sieve-like structure consisting of laminar beams, occupying the inner portion of the scleral sulcus, the groove circulating at the ICA. The SC is an adjoining, endothelium-lined, lymphatic-like circular channel that lies in the outer part of the scleral sulcus^5^. Usually, 20 to 30 CCs, 30 µm averaged in diameter with large variations can be found stemming from the SC to the aqueous veins in each human eye^6^.

Studies have found that juxtacanalicular tissue (JCT, which is the most outer layer of TM) and the inner wall of SC are the major sources of proximal outflow resistance that increase the IOP^4^. Several industrial corporations and research groups have introduced a variety of laser or non-laser devices to target and bypass the AH outflow resistance in the TM regions to restore the natural outflow pathway. These device inventions include Trabectome (NeoMedix, Tustin, CA, USA), Kahook dual blade (New World Medical, Rancho Cucamonga, CA, USA), iStent (Glaukos Corporation, San Clemente, CA, USA), Hydrus microstent (Ivantis Inc., Irvine, CA, USA), Argon laser trabeculoplasty^7^, excimer laser trabeculostomy^8^, selective laser trabeculoplasty^9^, and a recent reported prototype called femtosecond laser trabeculotomy (FLT)^10,11^ (ViaLase Inc., Aliso Viejo, CA, USA). In addition, it is intriguing to investigate the targeting location effect on the effectiveness of these trabecular surgeries; for example, surgical sites in the TM that are nearby the CCs might have higher IOP reduction than elsewhere far from the CCs. Therefore, visualization of the ICA details particularly the TM/SC/CC regions are strategically crucial for both clinical glaucoma treatments and research purposes.

Various optical and non-optical imaging methods have been developed for ICA imaging. Gonioscopy has been used for decades and is still the current gold standard for angle imaging^12^. By exploiting a goniolens, the light reflects off its interior mirror and travels through the cornea, reaching the surfaces of the angle structures, including the cornea, Schwalbe’s line, TM, scleral spur, iris, and sclera. However, these tissues’ depth information is not available on a gonioscopic image, and it produces results that are subjective and operator-dependent. A handheld camera, EyeCam (Clarity Medical Systems, Pleasanton, CA, USA), is also widely used for a similar purpose for angle imaging, but it shares the shortness with the aforementioned gonioscopy^13^. Ultrasound biomicroscopy (UBM) imaging is a contact procedure performed to visualize the angle with tissue penetration up to 5 mm at 25 µm axial resolution and 50 µm lateral resolution^14^. This imaging technology, however, is still far from resolving the TM/SC/CC details, particularly the CCs.

Optical coherence tomography (OCT) emerges as a new type of in-vivo, non-contact, micron-scale, three-dimensional imaging modality that has been widely used in ophthalmology^15^. Clinical studies using commercial OCT machines have shown the capability of the anterior segment (AS-) OCT for AC and ICA imaging. Typically they have an angle-to-angle scan covering the full or half range of the AC, including the cornea, limbus region, ICA, and sclera^16–21^. Imaging of the TM geometry has been widely investigated in clinical studies because this is usually the impeded site leading to reduced ocular outflow and glaucoma development^22–30^. The AS-OCT systems have also been used to measure the SC cross-sectional areas in healthy and glaucomatous patients^31–36^, or to study the SC morphological changes in response to different IOPs^37^ and glaucoma treatments^38–42^.

The commercialized AS-OCT systems however are not specifically designed or optimized for angle imaging and have lower axial and/or lateral resolutions. Several groups have built benchtop OCT systems or adopted a higher resolution OCT to visualize the TM, SC, and CCs by scanning the corneoscleral limbus region^43–48^. An early attempt by Kagemann et al. has shown the detailed angle structures via radial scanning at the limbus of the eye, but the deeper TM layers appears quite dim^43^. The same group has also demonstrated ‘virtual castings’ of the angle structures in both donor eyes^49^ and clinical subjects^50^. The basic operation relies on image intensity inversion, which might potentially mistake a common ‘shadow effect’ for the TM/SC/CC structures since both will represent as high-intensity signals on the intensity-inverted images. In addition, OCT variants have been introduced to visualize the structure and motion of the TM^44–46^. Recent advances of the angle imaging by the limbus scanning lie in the development of the full circumferential SC imaging, which requires multiple volumetric scanning and stitching^47,48^. Another way for angle imaging is by directing the OCT beam through the cornea, AH to the ICA at a relatively low angle. However, the backscattering light from the ICA structures experiencing the total internal reflection on the cornea makes this imaging technique challenging. Researchers have incorporated a custom design goniolens into an OCT device to circumvent this technological barrier^51,52^. Visibilities of TM and SC have been reported by these gonioscopic OCT systems, but the resolutions are still not sufficient to resolve the CCs. This is probably because the optical aberration issue worsens after layers of varying refractive indexes of gonioscopic optics, goniogels, and different ocular tissues through the AC at a low imaging angle. To sum up, image processing and hardware modifications in the field of iridocorneal angle imaging are still very limited, making visualization of the deep-seated TM/SC/CC challenging.

In this study, we presented a custom-built, high-resolution SD-OCT system to scan the corneoscleral limbus, and implemented different image processing for better visualization of the TM/SC/CC areas in human cadaver eyes. Simultaneously radial and circumferential scanning were implemented in the current system. A semi-automatic segmentation of the anterior corneal surface was carried out using a graph search algorithm and iterative method. Based on this segmentation, we further encoded the depth with ‘rainbow effect’ colors, which can be readily perceived by human eyes. In addition, intensity projections, and *en face* imaging were performed as supplemental information to the individual B-scan image and 3D reconstruction. To our best knowledge, this is the first time that the aforementioned novel or improved image processing and a ‘crossline’ scan have been implemented to achieve a better visualization and comprehensive understanding of the angle details. Future full integration of these imaging capabilities into an FLT system^10,11^ would potentially assist the accurate determination of the surgical targets and benefit other glaucoma treatments as well.

## Results

### Segmentation and depth encoding

**Figure 1:**
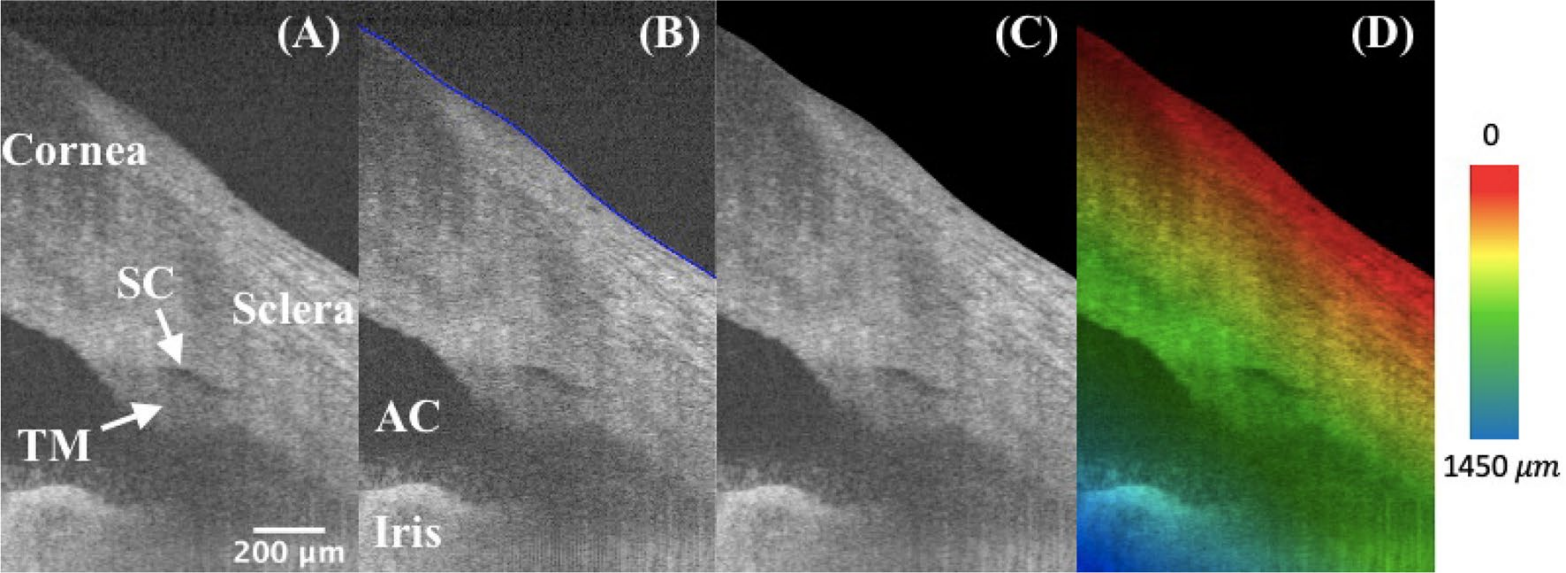
High-resolution iridocorneal angle OCT imaging, segmentation, and depth-encoding. (**A**) The example is an radial image, where the low-intensity prolate ellipse corresponds to the cross-section of a ring-shaped SC, which locates right above the TM. (**B**) The anterior surface was segmented and delineated in blue using a custom semi-automated algorithm. (**C**) The region outside of the surface boundary is blackened as zeros. (**D**) A depth-encoded image shows the rainbow effect starting from the segmented boundary into deeper tissues. TM: trabecular meshwork; SC: Schlemm’s canal; AC: anterior chamber.

Segmentation was performed on each B-scan image’s anterior surface of the eye before further processing. Figure 1A and B are typical OCT images before and after segmentation. The background region above the surface was masked out as shown in Fig. 1C. Figure 1D illustrates the rainbow effect of a depth color-coded image, in which starting from the segmented layer to the deeper tissue depths are displayed as a gradation of color from red, yellow, green, to blue. For example, the red color indicates that the tissue is shallow, and yellow indicates that the tissue is at a deeper location from the tissue’s anterior surface. The TM/SC/CC region is mainly within the green layer, and the iris is in the blue layer.

### Three-dimensional reconstruction and orthogonal viewing

**Figure 2:**
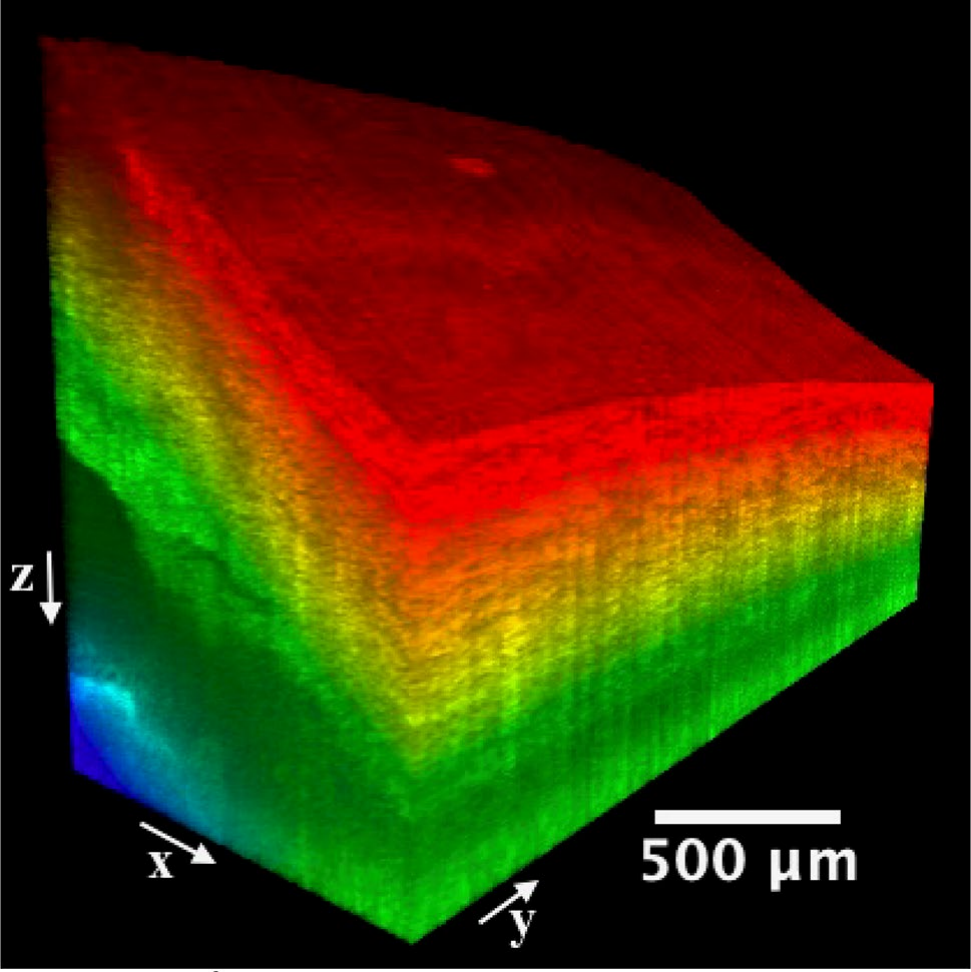
A 1.0 × 2.5 mm^2^ area at the iridocorneal angle of a human cadaver eye was scanned, resulting in a $1.0 \times 2.5 \times 1.6\;{\text{mm}}^{3}$ three-dimensional OCT cube. Included also the x, y, and z directions are defined and the origin is the vertex of the image cube on the top left (not shown). The colormap is coded along the imaging depth direction from the segmented layer on the surface. Same eye used in Fig. 1.

**Figure 3:**
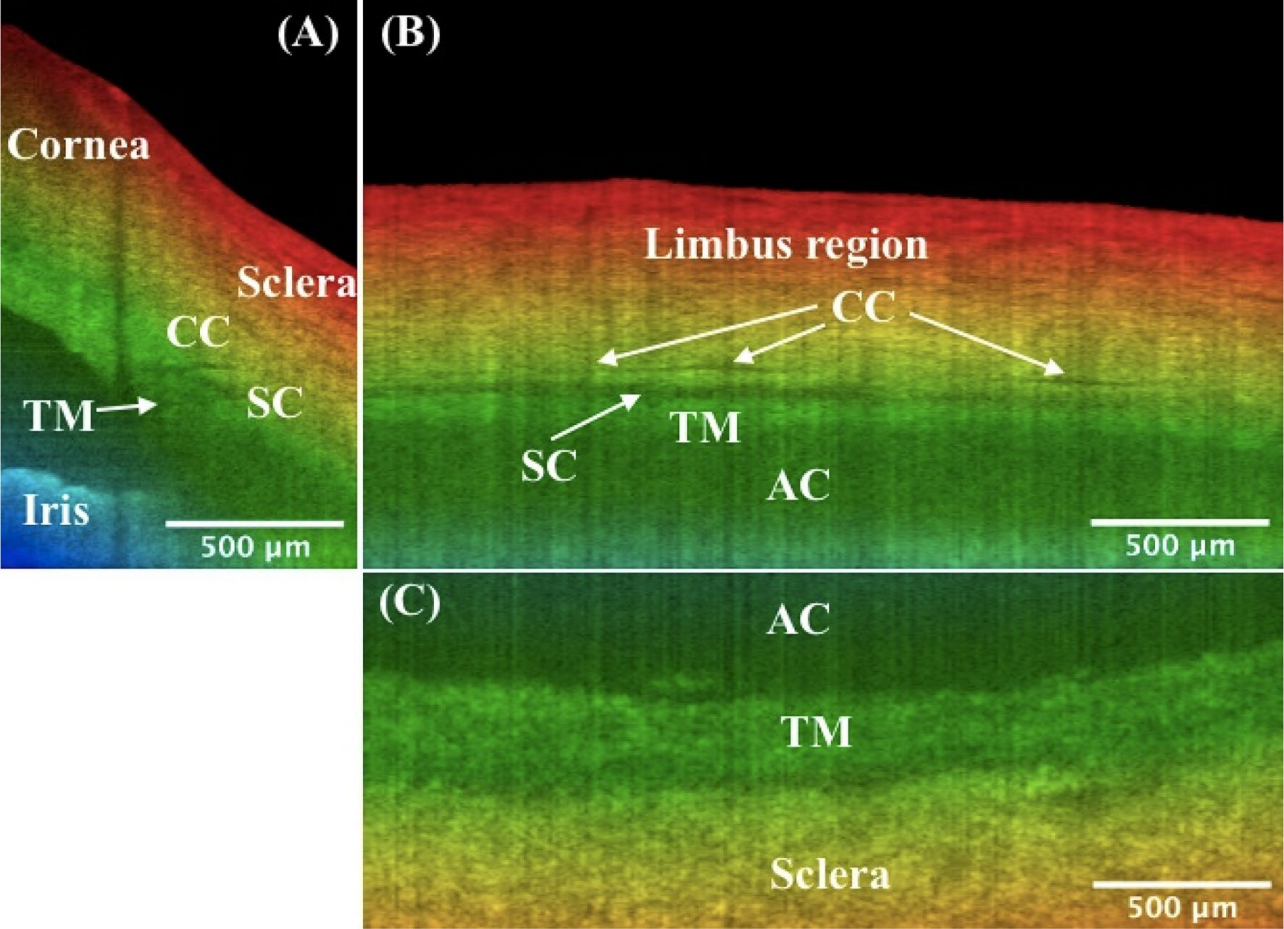
Orthogonal viewing by x-, y-, and z-slicing. (**A**) An xz-plane image reveals the SC’s cross-sectional area change and branching of CC stemming from the SC, as compared to Fig. 1D. Note shadow effect is evident which is cast by the damaged tissue on the tissue surface. (**B**) An x-slicing results in a yz-plane image, where the SC is clearly visualized as a low-intensity band right above the TM. Note CC is running approximately parallel above the SC. (**C**) A z-slicing through the center of SC from the top view allows for visualization of the juxtacanalicular tissue and inner wall of SC, which are considered the main source of resistance of the human ocular outflow. Also straightforward is that the TM locates at a relatively deeper depth than the nearby sclera. TM: trabecular meshwork; SC: Schlemm’s canal; CC: collector channel; AC: anterior chamber. More details such as the cross-sectional area changes of SC, distribution and branching of CCs, the morphology of TM/SC/CC region, and nearby vessels can be seen in video1_radialStack, and video2_3DSlice. Same eye used in Fig. 1.

The imaging capability of the proposed SD-OCT system for the iridocorneal angle in the limbus region was tested in a human cadaver eye study, as shown in Figs. 2 and 3. Figure 2 represents a 3D reconstruction of a stack of radial images, with the anterior surfaces segmented. An unintentionally damaged tissue was also found on the surface (Fig. 2), causing the shadow underneath along the depth direction (Fig. 3A). To reveal details in the TM/SC/CC region, orthogonal images can be obtained from the image volume by y-slicing (Fig. 3A), x-slicing (Fig. 3B), and z-slicing (Fig. 3C). Importantly, careful tracing of the continuous slices allows for revealing the details of the TM/SC structural changes and morphology of nearby CCs that are stemming from the SC and going circumferentially along the SC.

### Intensity projections

**Figure 4:**
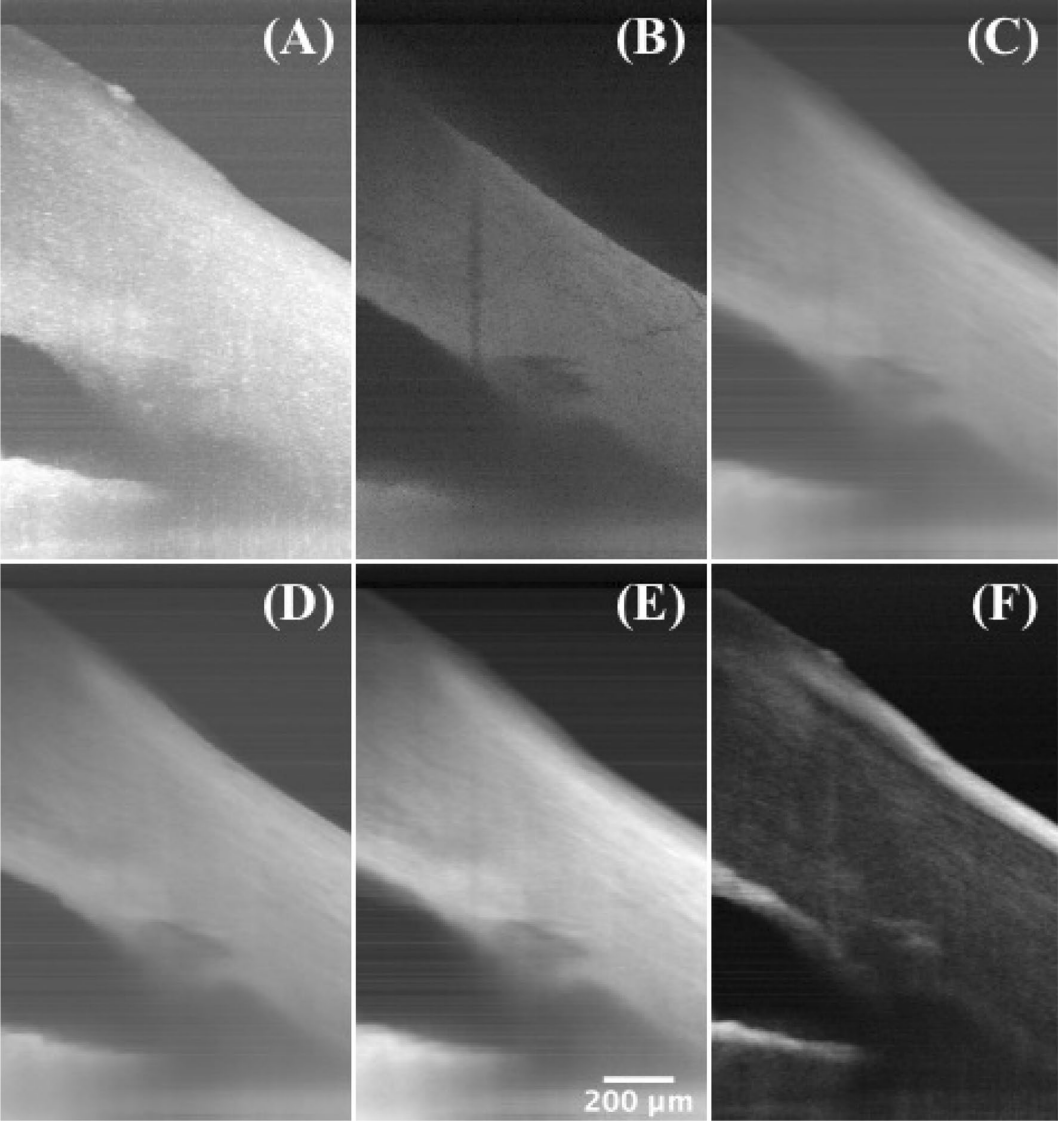
Intensity projections. (**A**) Maximum intensity projection, (**B**) minimum intensity projection, (**C**) average intensity projection, (**D**) median intensity projection, (**E**) sum intensity projection, and (**F**) standard deviation intensity projection along the y-axis of a 2.5-mm segment of radial scanning at the iridocorneal angle of a human cadaver eye, showing the TM/SC region and the damaged tissue on the surface. Same eye used in Fig. 1.

A series of projections using ImageJ software (National Institutes of Health, Bethesda, Maryland, USA) were performed to obtain more supplementary information. Figure 4A–F shows maximum intensity projection, minimum intensity projection, average intensity projection, median intensity projection, sum intensity projection, and standard deviation intensity projection, respectively, along the y-axis. The shadowing regions and the TM have much lower values on the minimum intensity projection, and the anterior and posterior surfaces of the corneoscleral tissue, and the surrounding regions of the SC show large variances on the standard deviation intensity projection.

### Enface imaging

**Figure 5:**
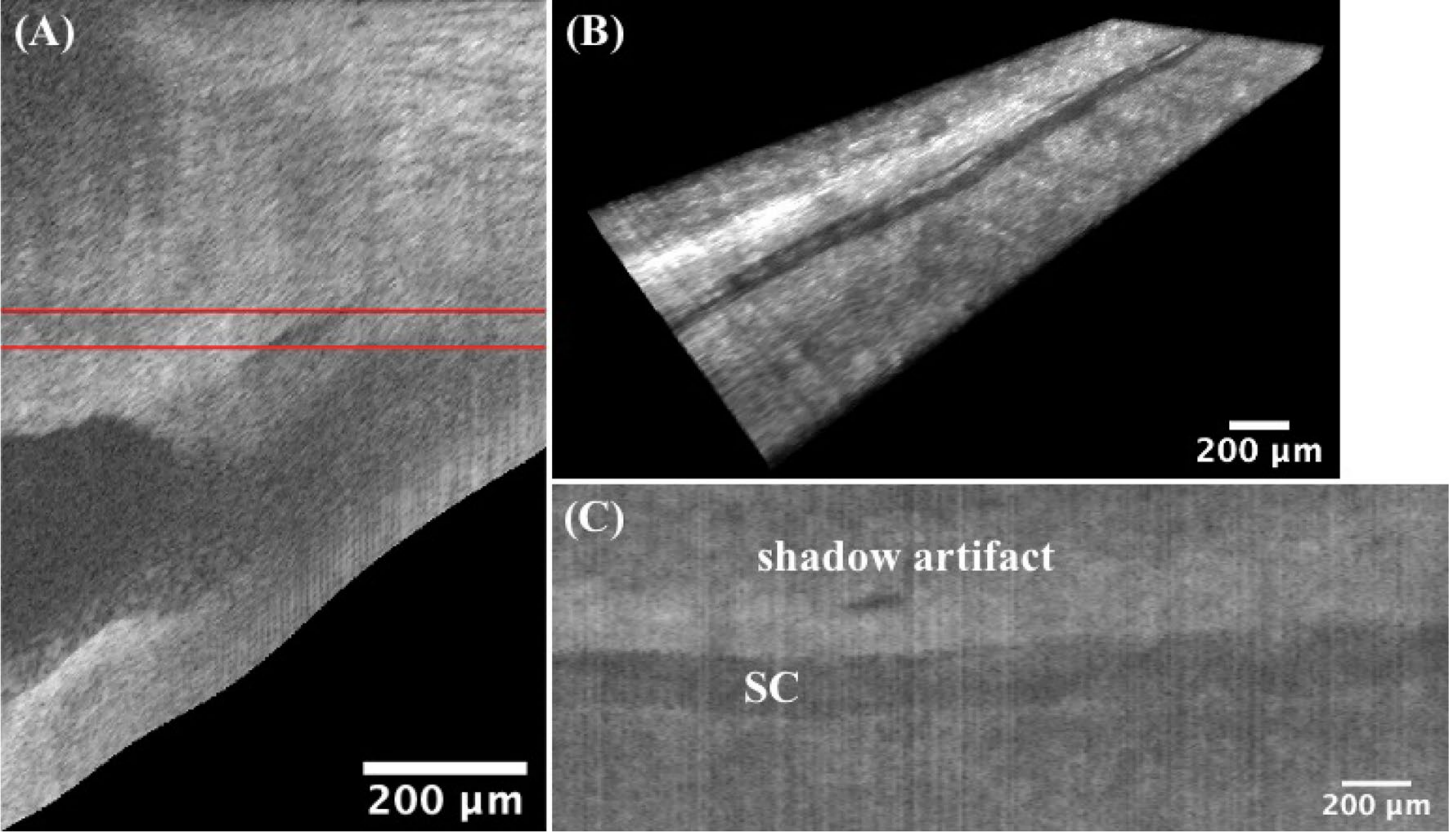
Enface Imaging. The image flattening technique is applied to each B-scan image along the anterior boundary for the entire scan volume. (**A**) shows only one representative flattened image of the whole flattened volume. (**B**) The thickness of the cropped flattened image cube is confined by the red lines (rows 286 and 320 in the flattened volume). (**C**) An SC region is shown on the minimum enface projection, which is performed along the depth direction on the image cube in B. The patent lumen of the SC is due to the relatively low backscattering in the fluid within the canal. Note the dark shadow artifact is caused by the damaged tissue on the surface. TM: trabecular meshwork; SC: Schlemm’s canal. Same eye used in Fig. 1.

After segmenting the anterior surface for the entire scan volume, images were flattened from the segmented boundaries so that the surface was in the first rows in the new image cube. Figure 5A shows one representative flattened image. By minimum enface projection along a depth range within the flattened imaging cube (Fig. 5B), an enface image was obtained, where the SC region was clearly visualized as a dark trough in Fig. 5C.

### Circumferential scanning

**Figure 6:**
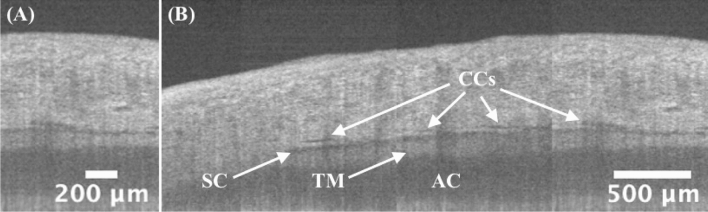
Circumferential scanning. (**A**) A typical circumferential image with details of TM/SC/CC shown. Some SC/CC narrow or discontinue but can be appearing larger at nearby radial locations (perpendicular to the paper direction), and this can be verified by 3D reconstruction of previous radial stacks. (**B**) A ‘panoramic’ view of TM/SC/CC regions, which is concatenated by non-overlapping, circumferential scans, demonstrating a 3.5-mm (i.e., 2.5-mm translation plus 1*-*mm image width itself) circumferential coverage of the iridocorneal angle. Continuous circumferential representation gives ophthalmologists direct access to the pattern change of the TM/SC/CC details, which is potentially applicable in preoperative planning for glaucoma surgeries. More dense and overlapping scanned images can be found in video3_cir. TM: trabecular meshwork; SC: Schlemm’s canal; CC: collector channel. Same eye used in Fig. 1.

**Figure 7:**
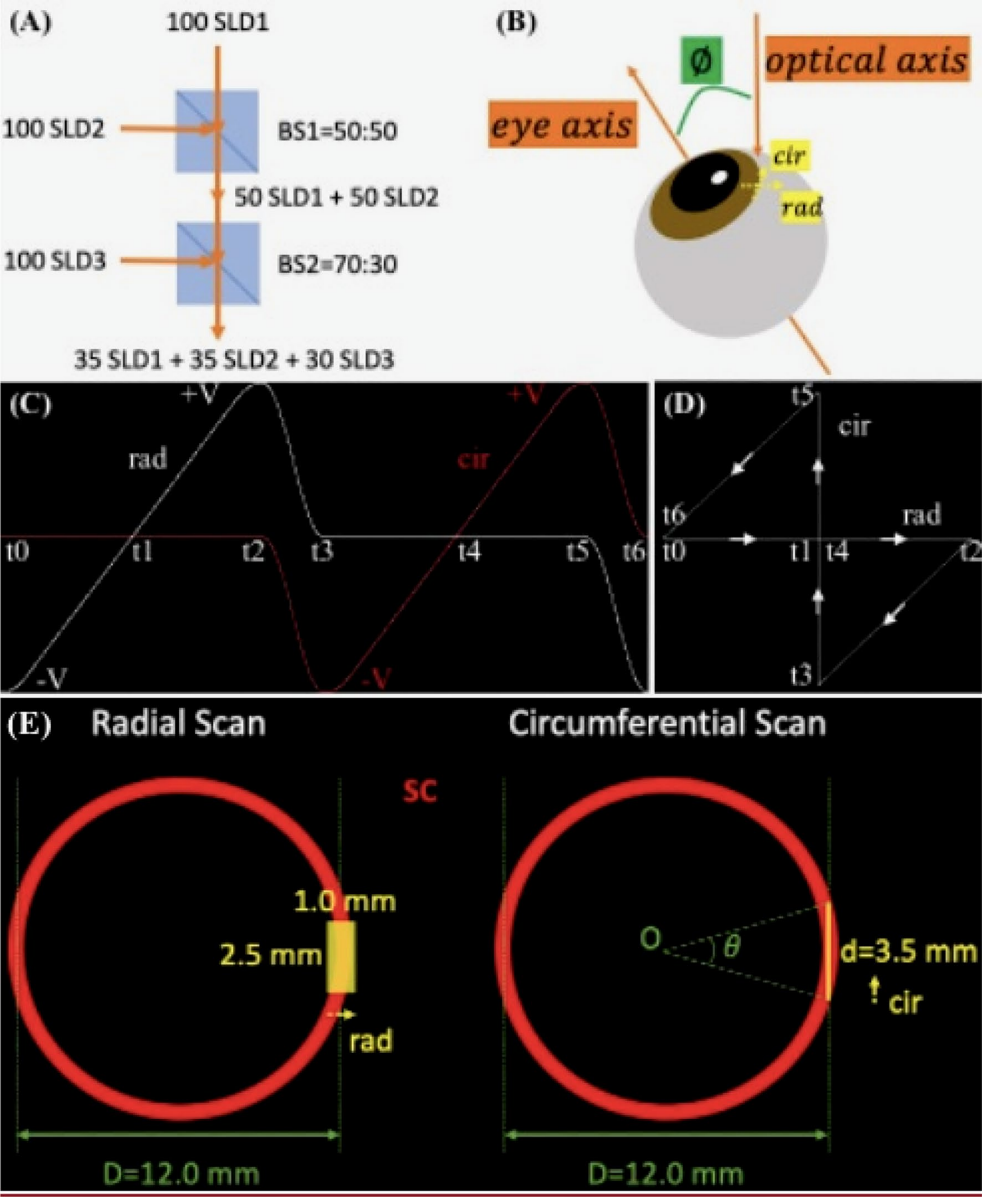
SD-OCT imaging and experimental setup. To obtain a higher axial resolution, (**A**) three SLDs were combined by two beam splitters, BS1 and BS2, to have a broader optical source spectrum. Assuming that the three SLDs each have 100 arbitrary units of spectral powers, after BS1 and BS2 with the respective transmission-to-reflection ratios of 50:50 and 70:30, the optical source output is generated by the combined spectrum which is consisted of 3 comparable spectra from each SLD. (**B**) The human cadaver eye is positioned on a translational stage (not shown) and is tilted at an off-eye axis angle for best imaging of the iridocorneal angle features. (**C**) The timing diagram of the crossline scanning for one period of radial and circumferential scans. Strictly speaking, the ‘simultaneous’ radial and circumferential scans are in fact working alternately. The galvonometer voltage controlling the circumferential scan flattens at zero when it scans radially, vice versa. (**D**) shows the corresponding 2D trajectory of the OCT beam at different time points in (**C**). Note our crossline (radial and circumferential) scanning is part of the bowtie-shaped track, where the two hypotenuses’ paths are used to shift between the two scans. In the projection view along the eye axis, a continuous 2.5 mm translation of the eye shown in B (normal to the paper towards readers) resulted in (**E**) a $1.0 \times 2.5\;{\text{mm}}^{2}$ radial scan and a 3.5-mm circumferential scan. The latter corresponds to a counter-clockwise, approximately $\theta = 2 \cdot \tan^{ - 1} \left( \frac{d}{D} \right) = 33^\circ$ of the full circumference of the angle structures, where D is the diameter of the ring-shaped SC estimated from reported averaged measurements of the spur-to-spur distance^57^. SLD: superluminescent diode; BS: beam splitter; rad: radial; cir: circumferential; SC: Schlemm’s canal.

Besides previous radially scanned images, circumferential images of the TM/SC/CC were simultaneously obtained by our custom-design crossline scanning at the limbus region (Fig. 7B–E). Figure 6A shows a circumferential image, and Fig. 6B is a concatenated circumferentially scanned image, providing a ‘panoramic’ view of TM/SC/CC region.

## Discussion

The vast majority of the ocular angle OCT imaging systems developed so far were designed for the whole anterior chamber (AC). These systems have a good characterization of the AC but have limited capability in resolving ICA details. An OCT system particularly for TM/SC/CC region is therefore imperative considering those important structures in the ocular outflow are associated with glaucoma development. In this study, we demonstrated the feasibility of an SD-OCT system custom-built for the ICA imaging. In addition, image processing algorithms were implemented to better visualize the angle details.

We used human cadaver eyes for the current OCT imaging verification, which was consistent with our previous glaucoma treatment studies in human donor eyes by the FLT surgical system^10,11^. Although mice are more readily available, their eye sizes are much smaller and the angle structures are different from human eyes^53^. Primates have the SC but they are extremely costly and involve more complex experimental protocols. Human cadaver eyes provide researchers the best access to studying the ICA details. The donor eyes from San Diego Eye Bank were within 24 h of death, allowing for preservation of cellular structure. In this study, the tissue structures of the eyes were kept intact, as opposed to our previous FLT surgery experiments. For best imaging of the TM/SC/CC details, a tilted angle between the OCT beams and the eye axis is required. In a clinical setting this can be accomplished by directing patients to focus on an external target on the side so that the OCT beams can impinge on the limbus region perpendicularly.

Since the depth encoding starts from the ocular surface, segmentation is needed before further operations. The graph search algorithm was previously reported and it was widely used in clinics^54^. Fully automated segmentation is possible but it is not robust for OCT images under different experimental scenarios. For example, the cornea side on the top left of the image has relatively low intensity and tends to be segmented erroneously by directly applying the graph search algorithm. A simple semi-automated segmentation framework can ensure the segmentation works more robustly and accurately within a restricted search region.

Depth color encoding allows for depth differentiation between TM/SC/CC and nearby structures. This would be beneficial to eye surgeons, for example, to distinguish the nearby structures, especially on the xy-plane images via z-slicing. Indeed, the JCT in a deeper depth (Fig. 3C, green) can be easily distinguished from the nearby sclera in a shallower depth (Fig. 3C, yellow). Note that the color gradient used in this work is only depth-encoded and it has nothing to do with segmentation and color assignment for different eye structures. While for the structures we are interested in this study, the sclera typically has a shallower location from the ocular surface than the deep-seated TM/SC/CC structures.

Three-dimensional reconstruction of radial image stacks and slice-by-slice orthogonal viewing allows for visualizing the TM/SC/CC region in more detail. Our previous study has used this imaging system to evaluate femtosecond laser trabeculotomy (FLT) channels in human cadaver anterior segments^10,11^. However, the sample tissues were imaged directly on a corneoscleral anterior segment artificially mounted such that the TM was directly facing the axis of the OCT beam. In this study, OCT light was irradiating the limbus region of an intact human cadaver eye to study the imaging capability of our prototype OCT system^55^. In addition, our previous study indicated that the TM geometry was not available when doing the FLT surgery so the outer wall of the SC might be unintentionally disturbed sometimes. Therefore, this work was to test the feasibility of imaging and quantifying the TM/SC/CC region in 3D. We have successfully detected the TM/SC/CC structures and distribution, showing a promise for presurgical planning for the FLT surgery. By using this imaging system, future work will utilize the 3D OCT to image guide the FLT glaucoma surgery and to evaluate the FLT channel creations and treatment efficacy in more detail.

Intensity projections and *en face* imaging are common techniques used in medical imaging which generate useful information. These views of images might provide supplementary information in location estimates of the target regions in an FLT or other glaucoma surgeries.

In addition, the simultaneously implemented circumferential images allow for visualization sweeping over the TM/SC/CC region circumferentially to get a panoramic view. Although 3D reconstruction of the radial image stacks can usually provide more detailed structures spatially, these serial 2D circumferential images can provide TM and SC, and the relative distributions of the CCs from the SC in real-time (video3_cir). This might help ophthalmologists get a quick idea of what the TM/SC/CC region looks like before a dense 3D volume scan is taken.

Several limitations should be addressed for cadaver eye or other animals’ ocular OCT iridocorneal imaging studies. Our current implementation of a volume scan was collected by radial image stacks by manually adjusting the translation for prototype verification. Future work can be integrating a fast 3D scan protocol. Second, the circumferential scanning length is limited due to the curved ring-shaped structures of the TM/SC circulating the cornea. To test the 3D imaging capability we showed a 2.5-mm scanning length circumferentially. The latest published techniques for 360° imaging of the SC employed the combination of multiple volumetric scanning^47,48,53^, which might cause discontinuity between volume scans. A larger field of view could be a solution but the resolution is limited and the direction of OCT beams is not optimal for the angle imaging. A potential solution to obtain the 360° angle structures could be the 3D reconstruction of a large number of radial scans in the TM/SC/CC regions. This will include further hardware modification for the imaging system and interpolation algorithm implementation for the angular spacing between adjacent radial B-scans. Lastly, for potential future clinical translation, the eye movement issue during the complete 360° ICA acquisition might be solved by the faster scanning technique.

In summary, our prototype was demonstrated to be able to visualize the ICA details. Both circumferential and radial images can provide researchers with the spatial representation of the TM/SC/CCs. A variety of image post processing have been implemented for better visualization of the angle details.

## Methods

### System setup

The SD-OCT system was described in our previous publication^55^. Briefly, our imaging system is working at 850 nm center wavelength with 165 nm bandwidth, which is combined by three superluminescent diodes (SLDs) (Fig. 7A). A pair of galvanometric scanning mirrors (Cambridge Technology Inc., Bedford, MA) was implemented which enables simultaneous radial and circumferential scanning of the iridocorneal angle of the eye (Fig. 7B–E). An objective lens that has an effective focal length of 54 mm (LSM54-850; Thorlabs, Newton, NJ) was used to focus the OCT beam onto the limbus region. Spectral interferograms are detected by a spectrometer (Cobra-S 800, Wasatch Photonics, NC), which are then Fourier transformed into B-scan images.

The line-scan camera has 2048 pixels and runs at a 20 kHz rate. Processed images contain 798 × 500 pixels per frame. The measured average axial resolution across 2-mm depths and theoretical lateral resolution are 2.7 µm and 8.2 µm in air, respectively. The physical lateral spacing is 2 µm. The OCT imaging system has a sensitivity of ∼110 dB and is capable of imaging the eye to a depth of approximately 1.5 mm with ∼4.0 *mW* incident light power on the sample. The power level remains below the maximum permissible exposure (MPE) limits for the retina, with the thermal MPE and photochemical MPE calculated to be 5.0 mW and 47.7 mW, respectively, for a scan time of 250 s^56^. Note that the OCT emission described in this paper is fully contained within the iridocorneal angle of the eye. No OCT light will be incident on the retina or the posterior segment of the eye.

### Sample preparation

The human cadaver eyes for OCT imaging were obtained from San Diego Eye Bank within 24-h postmortem. The study was in compliance with the Declaration of Helsinki. The eyes were stored in the Optisol corneal storage medium (Bausch and Lomb, Rochester, NY) and refrigerated at 4 °C, which kept the cells alive for several days. One eyeball was used in this study, and it was at good status without the need for any perfusion requirements. Before imaging, the eye was placed in an incubator (Sheldon Manufacturing, Inc., Cornelius, OR) at 37 °C, 5% CO_2_, and 90% humidity for 30 min. The eye was then placed on a customized eye holder on a movable mechanical stage with 5 degrees of freedom, i.e., X, Y, Z translations, rotation, and tilting under the OCT imaging system. The OCT beam was focused on the limbus to image the iridocorneal angle as illustrated in Fig. 7B. The cornea surface was kept moist to prevent dehydration during imaging. Note that the iris color is not known in this study, and the color shown in the figure is based on depth-encoding.

### Data acquisition and image preprocessing

Mirror data were collected for systematic dispersion compensation as described previously using time–frequency analysis and iterative optimization^55^. Before scanning the cadaver eye, background data were obtained by blocking the sample arm. We then decided on the scan region by finely tuning the translation stage’ vernier micrometers in both radial and circumferential directions to localize the TM/SC/CC region on both radial and circumferential images. In our experimental setup, we deliberately positioned the DC component, which represents the lower frequencies of the image, at the bottom of the image frames^58^. This arrangement was intended to optimize the visualization of angle details by enhancing their intensity. Three-dimensional tissue data were acquired at 10 µm intervals along a 2.5-mm distance circumferentially, which corresponded to a stack of radial images. A collection of circumferential data were generated simultaneously during this process.

These raw spectra data were read and processed in the MATLAB environment (MATLAB R2021b, MathWorks, Inc., Natick, MA) to generate B-can images. Specifically, the tissue spectra were firstly subtracted from the background, then uniformized by dividing by the normalized background which is within the 0 to 1 range. A Hann window was applied before Fourier transformation was done to generate the images. To avoid unresolved details approaching the Nyquist limit area, an up-sampling technique was used via $2\times$ zero-padding in the image space. Inverse Fourier transform of the padded images thus resulted in $2\times$ denser spectra, which were then k-linearized before dispersion compensation. Lastly, Fourier transform and logarithm operation was implemented to generate a gray-scale intensity B-scan image.

We assumed the averaged tissue refractive index as 1.336 of air, cornea, sclera, and aqueous humor for air-to-tissue scaling along the depth direction. Each B-scan image was then converted to isometric pixels using the ‘imresize’ function in MATLAB so that 2 µm per pixel was achieved. A $3\times 3$ median filter was applied, and the image was scaled to [0,1] before segmentation was done.

### Segmentation

**Figure 8:**
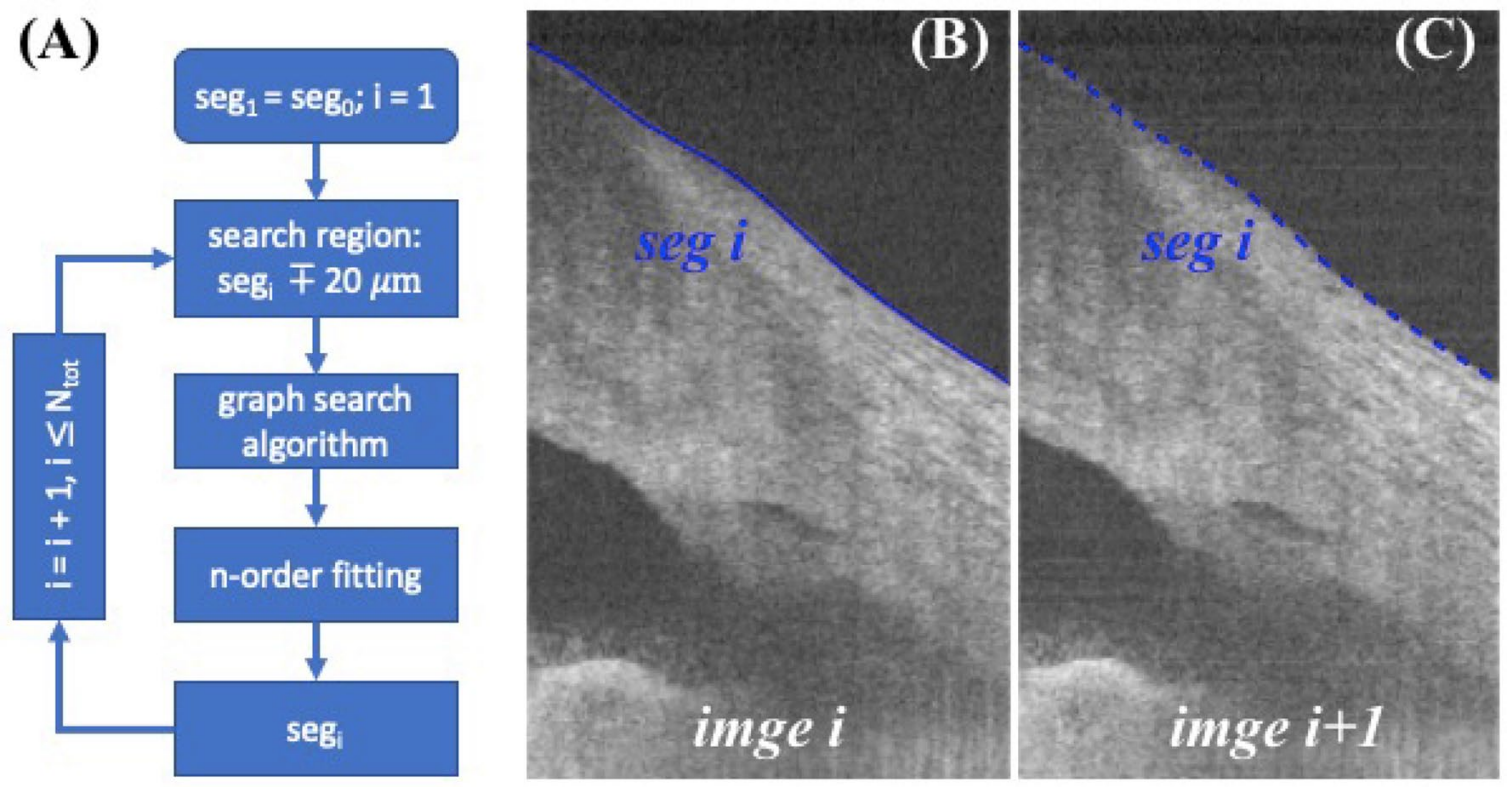
Segmentation framework. (**A**) Anterior surface image segmentation is initiated by manually segmenting the first image and iterating in a loop for the following B-scans in serial. The bottom block seg_i_ indicates the current image segmentation result and is used to determine the search region for the next B-scan’s image segmentation. (**B**) An example shows a typical image *i* with its segmented boundary *seg*_*i*_, and (**C**) image *i* + *1* with previous segmented result *seg*_*i*_ overlayed, which is used as an estimate to determine the search region by shifting ± 20 µm of *seg*_*i*_ for segmenting image *i* + *1*. For clarity segmentation result *seg*_*i*+*1*_ was not shown on (**C**) because it was close to *seg*_*i*_.

Segmentations of the anterior surface were performed slice-by-slice for the serial radial images by a graph-based algorithm and iterative method. Since the interval between B-scan images were only 10 µm separate in y-axis, we assumed that the segmented boundaries in adjacent images were within a small range, for example 20 µm. Initial segmentation was obtained by hand on the first image, image was flattened along this boundary and the search region was determined to be above and below 20 µm of the boundary. Gradient was calculated before applying the graph search algorithm to the current B-scan image. Segmented result was then fitted with a 9^rd^ ordering polynomial curve as the current segmentation result. Current segmented result was also used to determine the search region for the next B-scan image, and this process was repeated in a loop, as illustrated in Fig. 8 A-C. The proposed semi-automatic iterative method allows for fast segmentation, which requires only initial manual segmentation on the first B-scan and it is completely automatic for the remaining images.

### Image depth encoding

The color visualization was modeled in a Hue-Saturation-Value (HSV) color space. Specifically, the depth starting from the segmented layer was coded into the Hue channel, image intensity into the Value channel, and a constant, i.e., 1 into the Saturation channel. Note S, V values were normalized to the range in [0,1]; the H value was tailored from 0 to 2/3 so that a rainbow effect was achieved (changing from red, yellow, green, to blue). The HSV was then converted to RGB space for visualization and final image generation. Note the rainbow effect representation is available in FluoRender software (Scientific Computing and Imaging Institute, Salt Lake City, UT, USA), but it is incapable of encoding the depth starting from specific locations such as the segmented layer in our case.

### Image 3D reconstruction

Three-dimensional reconstruction was obtained by stacking the color images in ImageJ and loading them into FluoRender software for 3D visualization and slice-by-slice viewing.

### Supplementary Information

Supplementary Video 1.

Supplementary Video 2.

Supplementary Video 3.

## Data Availability

The data that support the findings of this study are available from the corresponding author upon reasonable request.
